# Gray Wolf (*Canis lupus italicus*) and Red Fox (*Vulpes vulpes*) Parasite Survey in Anthropized and Natural Areas of Central Italy

**DOI:** 10.3390/vetsci10020108

**Published:** 2023-02-02

**Authors:** Stefania Perrucci, Michela Maestrini, Francesca Coppola, Matteo Di Marco, Alessia Di Rosso, Maria Irene Pacini, Paola Zintu, Antonio Felicioli

**Affiliations:** Department of Veterinary Sciences, University of Pisa, Viale delle Piagge n. 2, 56124 Pisa, Italy

**Keywords:** wild canids, endoparasites, anthropized area, natural area, central Italy

## Abstract

**Simple Summary:**

Studies on wild animal parasites are considered crucial for the adoption of effective strategies aimed at reducing the impact of these pathogens on evolving ecosystems. This study aimed to assess and compare gastrointestinal nematodes and protozoa and other parasites detectable with coprological analysis in free-ranging wolf and red fox populations living in natural and anthropized areas of Tuscany (Central Italy). This comparison allowed us to detect significant differences in the occurrence and frequency of some parasite taxa considering the same canid species in different environments (natural and anthropized) and the two canid species in the same environment. Data obtained in this study may indicate different parasite risks and different roles played by the wolf and the fox in the diffusion of specific parasite taxa in the environments considered herein.

**Abstract:**

Gastrointestinal nematodes and protozoa and other parasite occurrences were evaluated in free-ranging wolf (*Canis lupus italicus*) and red fox (*Vulpes vulpes*) populations from natural and anthropized areas of Central Italy. Analyzed fecal samples were collected from 60 foxes and 40 wolves in the anthropized areas, and 41 foxes and 39 wolves in the natural areas. In foxes, hookworm infections (*p* < 0.0001) were more frequently recorded in the anthropized environment, while coccidia (*p* < 0.05) and *Cryptosporidium* spp. (*p* < 0.0001) were more frequent in the natural area. In wolves, a higher frequency of hookworms (*p* < 0.0001) was observed in natural areas, while coccidia were more common in the anthropized area (*p* < 0.05). Moreover, in the natural environment, trichuroid nematodes (*p* < 0.0001) were significantly more frequent in wolves than in foxes, while *Cryptosporidium* (*p* < 0.001) and *Giardia duodenalis* (*p* < 0.001) were more common in foxes. In the anthropic area, the occurrence of hookworms was found to be significantly higher in foxes (*p* < 0.0001), while trichuroid nematodes were more common in wolves (*p* < 0.0001). The obtained data are indicative of a different diffusion of specific parasite taxa in wolves and foxes living in the natural and/or anthropized environments examined herein.

## 1. Introduction

Wildlife parasites are considered an important field of investigation as they may have a significant impact on the health, dynamics, and sustainability of wildlife populations [1]. Ecological factors, combined with environmental changes mainly resulting from human activities, may contribute to amplifying parasite risk for wildlife and the risk of parasite transmission between wildlife, domestic animals, and humans [2,3,4]. Both the expansion of human activities and the increasing presence of wildlife in anthropized areas have strongly increased the possibilities of contact between wildlife, domestic animals, and humans and the risk of parasites spreading from wild to domestic cycles [2,3]. In Italy, over the last few years, there has been a reduction in natural habitats, causing the urbanization of wildlife, associated with a population increase in some species, such as wolves and ungulates [5,6]. 

Data on wild animal parasites are considered crucial for the adoption of effective strategies aimed at reducing the impact of parasites on evolving ecosystems. Furthermore, wildlife is recognized as an important source of human parasites, which may have a significant potential impact on human health [2,3,4,7]. The canid–human relationship is a typical example of animal–human coevolution since canids are the oldest and most widespread family worldwide and show high diversification across time and space. Several ecological factors may limit human–canid coevolution, influencing their mutual benefit, such as pathogen exposure [8]. Among wild hosts, canids have been identified as the most frequent source of parasitic infections in humans [7]. Moreover, foxes (*Vulpes vulpes*), wolves (*Canis lupus italicus*), and domestic dogs share several endoparasite species, including zoonotic nematodes and protozoa [2,3]. 

Therefore, data on these endoparasites in fox and wolf populations living in anthropized and natural areas could provide useful information for public health and wildlife ecology, as well as the dynamic of these parasites in wild and anthropic environments in a one health approach. However, to the best of our knowledge, in Italy, there are no previous studies comparing gastrointestinal parasites in wolves and foxes living in different environments.

This study aimed to evaluate and compare gastrointestinal nematodes and protozoa and other parasites detectable by using coprological analysis on wolf and red fox populations living in natural and anthropized areas of Tuscany (Central Italy). 

## 2. Materials and Methods

### 2.1. Study Sites 

**Figure 1:**
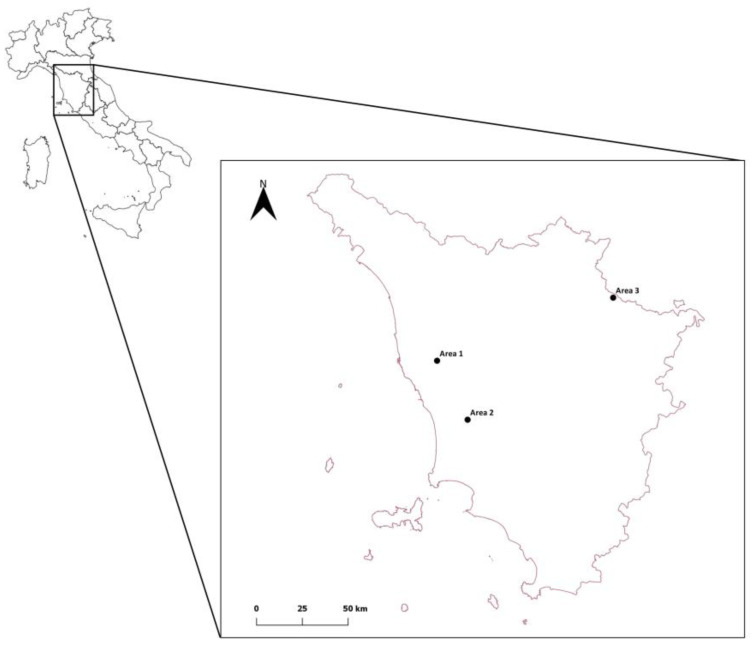
Map of the location of the Tuscany Region. In detail, the location of the anthropized site in Pisa Province (Area 1), the Monterufoli Caselli Nature Reserve (Area 2), and the Foreste Casentinesi National Park (Area 3) (black dots), where sampling was performed.

Samples were collected from three different areas, including two natural sites and one anthropized site located in Tuscany, Central Italy (Figure 1). 

The anthropized site (Area 1) is located in Pisa Province within the municipalities of Crespina Lorenzana and Casciana Terme Lari (10.56815° N–43.56796° E) and includes 18 towns, with an average human density of 134.08 people/km^2^. The area is characterized by a highly anthropized fragmented agroecosystem in which small woody areas are interspersed with agricultural and urban zones [9]. A wide variety of wild mammals live in this area, such as crested porcupines (*Hystrix cristata*), wild boars (*Sus scrofa*), roe deer (*Capreolus capreolus*), pine martens (*Martes martes*), stone martens (*Martes foina*), skunks (*Mustela putorius*), badgers (*Meles meles*), hares (*Lepus europeus*), eastern cottontails (*Sylvilagus floridanus*), wild rabbits (*Oryctolagus cuniculus*), red foxes (*Vulpes vulpes*), wolves (*Canis lupus*), and the introduced invasive coypu (*Myocastor coypus*) [10].

The natural area includes two different sites: the Monterufoli Caselli Nature Reserve of (Val di Cecina, Pisa, Central Italy, 43°15′6.48″ N, 10°45′26.64″ E) (Area 2) and the Foreste Casentinesi National Park (43°50′36″ N 11°47′28″ E) (Area 3). The Monterufoli Caselli Nature Reserve covers an area of 4.978 ha and is characterized by wide woody areas of Mediterranean scrub and oaks (*Quercus ilex)*. Hares; wild ungulates (mainly wild boars, fallow deer (*Dama dama*), and mouflons (*Ovis musimon*)); and carnivores such as red foxes, wolves, pine martens, weasels (*Mustela nivalis*), badgers, stone martens, and wild cats (*Felis silvestris silvestris*) are the most representative wild mammals in the area [11]. The Foreste Casentinesi National Park (43°50′36″ N 11°47′28″ E), which extends along the Tuscan-Romagna Apennine Ridge [12], is characterized by a great richness and variety of wild fauna. The most common mammals are the red deer (*Cervus elaphus*), the fallow deer, the roe deer, wild boars, mouflons, and the wolf, the largest predator present in the park. At least 21 species of micro- and meso-mammals have been observed in the park territory, among which the most common are the fox, the wild cat, the hare, the European mole (*Talpa europaea*), the blind mole (*Talpa caeca*), the red squirrel (*Sciurus vulgaris*), the crested porcupine, and the raccoon (*Procyon lotor*), as well as several mustelid species, such as badgers, weasels, stone martens, skunks, and martens.

### 2.2. Sampling

Wolf and fox fecal samples were collected along transects. In each sampling area, two transects (2 SD 1.3 km) were randomly chosen and were covered monthly. Only samples judged fresh were collected for parasitological analysis. The freshness state of fecal samples was determined based on external appearance and local weather conditions.

Fox and wolf fecal samples were distinguished according to their morphology, size, shape, smell, and content [13,14,15].

Collected samples were frozen at −20 °C until parasitological examination. 

### 2.3. Parasitological Analysis

For the detection and quantification of nematode eggs and coccidian oocysts, all the fecal samples were examined by using the flotation test and Mini-FLOTAC technique on 2 g of feces. Saturated sodium chloride (NaCl; specific gravity, 1.2) was used as the flotation solution [16].

Moreover, all fecal samples were examined by using a commercial rapid immunoassay to search for *Giardia* and *Cryptosporidium* spp. fecal antigens (Rida Quick^®^ Cryptosporidium/Giardia Combi, R-Biopharm, Darmstadt, Germany). 

Magnifications of 100× and 400× were used to identify nematode eggs/larvae and protozoan oocysts/cysts, which were measured under an optical microscope by using a micrometric eyepiece. Microscopic parasite identification was based on morphological and metric data [17,18,19,20,21,22]. 

### 2.4. Statistical Analysis

Data were processed using JMP 7 software (SAS Institute, 2008, Cary, NC, USA). Differences in parasite taxa frequency between wolf and fox as well as in natural vs. anthropized areas were analyzed using the X^2^ test, while Student’s *t*-test was used for the quantitative data analysis for wolves and red foxes in different habitat types. *p*-values lower than 0.05 were considered significant.

## 3. Results

A total of 180 fecal samples were collected and analyzed, of which 79 were from wolves and 101 from red foxes. In the anthropized area, 100 fecal samples were collected, while 80 were collected in the natural area (Table 1).

Overall, in the natural environment, 77.5% (62/80) of the examined wild canid fecal samples were found to be positive for at least one selected parasite species, with 78.05% (32/41) of fox and 76.9% (30/39) of wolf samples resulting in positives. The identified parasites were Ancylostomatidae (hookworms, *Uncinaria stenocephala*, and *Ancylostoma caninum*), Trichuroidea (*Eucoleus aerophilus*, *Eucoleus boehmi*, *Aonchoteca putorii*, and *Trichuris vulpis*), coccidia (*Isospora* spp., *Cystoisospora ohiensis* complex, and *Cystoisospora canis*), ascarids (*Toxocara canis* and *Toxascaris leonina*), *Cryptosporidium* spp., *Giardia duodenalis*, *Spirocerca* spp., *Entamoeba* spp., *Balantidium coli*, and *Physaloptera* spp. (Table 2).

*T. leonina* was found only in two ascarid-positive wolf samples, while *Spirocerca* spp., *B. coli*, and *Physaloptera* spp. were found only in fox samples. 

Of the wolf samples, 64% (25/39) were positive for one (38.46%, 15/39) or two parasite species (25.64%, 10/39), while 10.25% (4/39) were positive for three parasites. Hookworms, trichuroid nematodes, coccidia, and/or ascarids were the most frequent concurrent parasites in wolf samples. Among fox samples, 43.9% (18/41) were positive for a single parasite species, i.e., hookworms or trichuroid nematodes, but 19.5% (8/41) of samples were found to be positive for four or more species, mainly represented by hookworms, trichuroid nematodes, coccidia, and ascarids/*Cryptosporidium* spp./*G. duodenalis*.

Statistical differences were recorded in the frequencies of the identified parasites (X^2^ = 191.39, *p* < 0.0001), with ancylostomatid and trichuroid nematodes found to be significantly more frequent than all other parasites (Table 2).

Comparing the frequencies of identified parasites in the two canid species, the frequency of trichuroid nematodes (X^2^ = 15.044; *p* < 0.0001) was significantly higher in wolves than in foxes. Conversely, *Cryptosporidium* spp. (X^2^ = 12.084; *p* < 0.001) and *G. duodenalis* (X^2^ = 6.797; *p* < 0.001) were significantly more frequent in foxes than wolves (Table 3).

In the anthropized area, 87% (87/100) of the total analyzed wolf and fox fecal samples were found to be positive for at least one parasite species, with 91.7% (55/60) of fox and 80% (32/40) of wolf samples being positive. Overall, parasites detected in this area were the same as those found in wolves and foxes in the natural area, except for the nematode *S. stercoralis* found in a single fox (Table 2 and Table 3). 

In the anthropized area, 70% (28/40) of wolf samples were found to be positive for one (40%, 16/40) or two parasite species (30%, 12/40), and only 2.5% of wolf samples (1/40) were positive for three parasites. Trichuroidea and coccidia or ascarids were the most frequent concurrent parasites found in wolf samples. Among fox samples, 48.3% (29/60) were positive for one (17/60, 28.3%) or two (12/60, 20%) parasite taxa; the latter were represented by an association between hookworms and trichuroid nematodes, coccidia, or ascarids, but 11.7% (7/60) of the samples were found to be positive for hookworms associated with two or more species.

Overall, the occurrence frequencies of the different parasite taxa detected in the anthropized area were significantly different (X^2^ = 289.158; *p* < 0.0001). As observed in the natural area, hookworms and trichuroid nematodes were the most frequently detected parasites (Table 2). However, the occurrence of hookworms was higher in foxes compared with wolves (X^2^ = 119.66; *p* < 0.0001), while trichuroid nematodes were significantly more common in wolves than in foxes (X^2^ = 19.087; *p* < 0.0001) (Table 3). No significant differences were recorded in the occurrence of ascarid, *G. duodenalis*, and *Entamoeba* spp. between wolves and foxes (Table 3).

In comparing the natural and anthropized environments, only *Cryptosporidium* spp. (X^2^ = 11.898, *p* < 0.001) was significantly more frequently detected in natural areas compared with the anthropized ones. In foxes, hookworm infection (X^2^ = 20.802; *p* < 0.0001) was most frequently recorded in the anthropized environment. However, a similar number of eggs per gram of feces (EPG) for these parasites was counted in foxes from natural and anthropized areas. The frequency of coccidia (X^2^ = 7.167; *p* < 0.05) and *Cryptosporidium* spp. infections (X^2^ = 19.709; *p* < 0.0001) was higher in the natural area than in the anthropized one, and a higher number of coccidian oocysts per gram of feces (OPG) was also counted in the natural area. Conversely, the number of ascarid EPG counted was higher in the anthropized area (Table 4).

In both areas, the most common trichuroid nematodes in wolf and fox samples were the respiratory species *E. aerophilus* and *E. boehmi*. *T. vulpis* eggs were identified only in four wolf samples and one fox fecal sample, which were found to be positive for trichuroid eggs in the natural area, and in one wolf and one fox sample in the anthropized area, always concurring with *E. aerophilus* and/or *E. boehmi*. The gastrointestinal capillariid species *A. putorii* was also found in two fox samples from the natural area and in one fox fecal sample from the anthropized one. Among hookworms, all wolf samples were found to be positive only for *U. stenocephala*, with an egg length exceeding 80 µm. In fox fecal samples, positivity for *A. caninum* was also evidenced in two samples from the anthropized area, concurring with *U. stenocephala*. Furthermore, among coccidia, the species *Cystoisospora ohiensis* complex, *Cystoisospora canis*, and *Sarcocystis* spp. were detected in wolves from both areas, while *Neospora*/*Hammondia* spp. oocysts were only found in one wolf sample from the anthropized area. In foxes, *Isospora* spp. were recorded in both areas.

## 4. Discussion

This study assessed and compared gastrointestinal nematodes and protozoa and other parasites in free-ranging wolf and fox populations living in natural and anthropized areas.

In both the natural and anthropized areas, the same parasite taxa were recorded in wolf and red fox fecal samples, except for *S. stercoralis*, which was found in a red fox in the anthropized environment. However, the two wild canid species showed differences in the number of parasite taxa detected in relation to the environmental type, and the red fox was found to host a larger number of parasites among those investigated. In both environments, hookworms and trichuroid nematodes were the most frequently recorded parasites in the examined samples. This result confirms previous data evidencing a high frequency of hookworm and trichuroid infections in red foxes and wolves in Italy and other European countries [23,24,25,26,27]. All wolf fecal samples resulted in positives only for *U. stenocephala* species, in accordance with previous studies [23,24,25], while in fox samples, also positivity for *A. caninum* was evidenced in a few samples and always concurrently with positivity for *U. stenocephala*. Both *U. stenocephala* and *A. caninum* can also infect domestic dogs, and *U. stenocephala* can infect domestic cats [17,18]. *A. caninum* is considered a zoonotic species, as third-stage larvae contaminating the environment can be responsible for cutaneous *larva migrans* in humans [28]. The results showed that the frequencies of hookworm occurrence in the two wild canid species significantly differ according to habitat type. Although no differences were found in the frequency of hookworms between foxes and wolves in the natural environment, in the anthropized environment, these nematodes were more frequent in foxes than in wolves. Moreover, within each canid species, these parasites were found to be significantly more frequent in wolves living in the natural area. Conversely, hookworm frequency in foxes was significantly higher in the anthropized area compared with the natural environment. These differences may be due to both ecological and biological factors. The higher detection rate of Ancylostomatidae in wolves in natural areas may depend on the higher suitability of these environments. The natural area is characterized by higher moisture and more wooded areas, which are ideal for the development and survival of eggs and larvae [21]. At the same time, the high occurrence of these parasites in foxes in both natural and anthropic environments could be related to the feeding habits of this canid species. Unlike the wolf, which is a selective generalist predator at the top of the food chain [29,30], the fox shows much more opportunistic feeding behavior, based on small mammals, birds, invertebrates, and fruits [31]. The fox’s feeding behavior and predation of small animals potentially acting as paratenic hosts [21] may expose this canid to a high risk of hookworm infections in both natural and anthropic environments. Therefore, the results suggest that the probability and risk of hookworm infection in these two wild canids could be affected by both environmental and etho-ecological cues. Therefore, in epidemiological studies, foxes and wolves should be indifferently selected in natural areas as indicators of these important parasites, while in anthropic environments, only foxes should be considered. However, further studies are needed to confirm the usefulness of this approach.

Along with hookworms, trichuroid nematodes were also found to be the most frequent parasites in both investigated areas. Unlike Ancylostomatidae, no differences were recorded regarding the occurrence of trichuroid nematodes within wolves or foxes in the different habitat types. In accordance with previous studies, in both wolves and foxes, [24,32,33,34] respiratory capillariids (i.e., *E. aerophilus* and *E. boehmi*) were the most frequently recorded trichuroid nematodes compared with the intestinal species *T. vulpis* and *A. putorii*. Trichuroid nematodes may cause respiratory and gastrointestinal distress of varying severity, and in infected animals, they may reduce their hunting ability, as reported in domestic dogs [17,18]. Although few human clinical cases have been reported, *E. aerophilus* is considered a zoonotic nematode species [35]. *E. aerophilus*, *E. boehmi*, and *T. vulpis* are common in domestic dogs, while infections caused by *E. aerophilus* and *A. putorii* have also been reported in cats in Italy [36,37].

In Europe, the fox is considered the main reservoir for *Eucoleus* spp. infections in domestic carnivores [38]. However, the frequency of *Eucoleus* spp. detected in this investigation was significantly higher in wolves than in foxes in both environments, and a higher mean EPG number was found in wolves in the natural environment. Therefore, wolf-mediated environmental contamination may represent a high risk of capillariid infections in other animals, such as domestic dogs, both in natural and anthropic environments. Moreover, these results indicate that wolves may be a more reliable indicator of the spread of these parasites than foxes in both natural and anthropic environments, at least in the investigated area. 

The frequency of ascarid eggs found in this study in fox and wolf fecal samples was, overall, much lower compared with the abovementioned nematodes. However, no statistical differences in ascarid occurrences were recorded between these carnivores in the same habitat type and between different habitats in each wild canid species. *T. canis* was the most frequently recorded species infecting both foxes and wolves, while *T. leonina* was only recorded in a few wolf samples. These data confirm some previous observations showing a low/medium prevalence of *T. canis* and a very low prevalence of *T. leonina* in these wild canids in Italy and other European countries [23,27]. However, a higher sensitivity of necropsy than copro-microscopical analysis for the detection of ascarid infections has been reported [32]. Both these pathogenic species infect the domestic dog, while the cat is infected only by *T. leonina* [17]. Moreover, many animals may act as paratenic hosts, such as birds and mammals, while invertebrates can act as transport hosts [39]. Humans may also be infected when ingesting mature *T. canis* eggs containing infective larvae that can be responsible for human visceral, ocular, and cerebral *larva migrans* [40,41].

An interesting observation was the presence of *Spirocerca* nematode eggs in fox fecal samples from both natural and anthropized areas, rarely reported in Italy [33,42,43,44]. Moreover, until recently, spirocercosis, a disease that can be fatal in dogs and wild canids, was thought to be caused exclusively by *Spirocerca lupi* [43]. However, a new species, *Spirocerca vulpis*, was recently described in the red foxes, differing from *S. lupi* based on morphometric and molecular analysis [43], and its presence has been reported in several European countries, including Italy [43,44]. Therefore, the species found in foxes could be attributable to *S. vulpis*, but molecular analyses are needed for the identification of this species.

*Physaloptera* spp. gastric nematodes have been reported in Italy both in foxes and wolves with low/medium prevalence [20,23,33]. In this study, *Physaloptera* was found at very low frequency and only in fox samples from both natural and anthropized areas. *Physaloptera* spp. can infect the domestic dog, which can cause gastritis, vomiting, and weight loss [45]. However, little is known about the pathogenic role of these parasites in wildlife [20]. The species *P. sibirica* has been identified in foxes and wolves in the northwestern regions of Italy [20,23]. Again, differences in the diets of wolves and foxes may mean there is a higher risk of infections in foxes than in wolves in the examined areas, considering that insects act as intermediate hosts for *Physaloptera* spp. [20].

A single fox sample collected in the anthropized area was found to be positive for *S. stercoralis*, a nematode species that infects canids and primates but is very rarely reported in the red fox [46].

Among protozoa, the detection of *G. duodenalis* and *Cryptosporidium* spp. in this study should be emphasized since data on their diffusion in fox and wolf populations in Europe, including Italy, are very limited [47,48,49,50]. In the wolf and fox, these protozoa have been frequently reported at low prevalence [47,48,49,50]. Conversely, in this study, *G. duodenalis* and *Cryptosporidium* spp. were, overall, detected at high frequency. Nevertheless, in some studies, frequencies comparable to those found in this study have been observed, such as *G. duodenalis* infections in wolves in Poland and Croatia [51] and *Cryptosporidium* spp. infections in red foxes in Spain [52]. These protozoa are potentially zoonotic [52], and the diffusion of zoonotic genotypes in anthropized and wild environments may be the potential cause of infections in humans other than in wild and domestic animals. Genotyping these protozoa has shown that foxes and wolves are frequently infected by potentially zoonotic *G. duodenalis* assemblages and *Cryptosporidium* species, such as *C. parvum*, *C. ubiquitum*, and *G. duodenalis* assemblage A [52,53]. Therefore, the results obtained in this study are worthy of further investigations aimed at identifying the genotypes that occur in wild canids in the examined areas. Although detected in samples from both wild canid species and in natural and anthropized areas, these protozoa were more frequently found in fox samples than in wolf samples and showed a significantly higher frequency in natural areas. These results confirm previous data showing a higher frequency of *G. duodenalis* and *Cryptosporidium* spp. in natural environments and in foxes than in other wild carnivores, such as wolves [52]. 

Regarding coccidia, *Isospora*-like oocysts were identified in fox samples of both areas, confirming previous observations performed on foxes in Italy and other European countries [54,55]. The detection in this study of a higher frequency and intensity of infections in the natural environment may be linked with a higher number of juvenile fox fecal samples collected in this area, as these protozoa are prevalent among young animals [21]. The identification of *C. canis*, *C. ohiensis* complex, and *Sarcocystis* spp. in wolf samples in both areas, even if mainly in the anthropic area, and *Hammondia*/*Neospora* oocysts in a wolf sample from the anthropized area confirms previous observations on the presence of these protozoa in Europe [47,56]. Coccidia can cause severe infections in canids, especially in pups, or in association with other stress factors [57]. Therefore, these results underline the role of wild canids as possible sources of infection for dogs and other domestic and wild animals, acting as intermediate or paratenic hosts for these species. 

Although reported in wild canids in previous studies [58,59], the significance of detecting amoebic and *Balantidium coli* cysts in very few wolf and fox samples in this study is not known.

## 5. Conclusions

This study is the first example of the potential use of wild canids in comparing occurrences of gastrointestinal nematodes and protozoa and other parasites in anthropic and natural environments. The monitoring of parasites and the evaluation of their interactions with different environments can contribute to the maintenance of healthy ecosystems, and it can be used to assess the potential parasite risk for wild populations in different environments and the risk of parasite transmission between wild and domestic species and to humans [57]. In this context, the results may indicate different parasite risks and different roles played by the red fox and wolf in the diffusion of the investigated parasites, mainly hookworms, trichuroid nematodes, and *Cryptosporidium* spp., in the anthropic and natural environments examined in this study. 

## Figures and Tables

**Table 1:** Number of red fox and gray wolf fecal samples collected in anthropized and natural areas from Tuscany (Central Italy) and examined for parasites.

Anthropized Area		
**Samples**	**Fox**	**Wolf**
	60	40
Total samples	100	
**Natural Area**		
**Samples**	**Fox**	**Wolf**
	41	39
Total samples	80	

**Table 2:** Overall frequencies of parasites identified in wild canid fecal samples in the natural and anthropized area. Significant differences recorded for parasite occurrence within the natural and anthropized areas are indicated with different superscript letters.

Parasite	Frequency (%)	
	Natural Area	Anthropized Area
Ancylostomatidae	42/80 (52.2) ^a^	51/100 (51) ^a^
Trichuroidea	38/80 (47.5) ^a^	44/100 (44) ^a^
Coccidia	18/80 (22.5) ^b^	20/100 (20) ^b^
Ascarids	9/80 (11.25) ^b^	9/100 (9) ^c^
*Spirocerca* spp.	1/80 (1.25) ^c^	5/100 (5) ^c^
*Giardia duodenalis*	6/80 (7.5) ^b^	5/100 (5) ^c^
*Entamoeba* spp.	1/80 (1.25) ^c^	3/100 (3) ^c^
*Balantidium coli*	1/80 (1.25) ^c^	2/100 (2) ^c^
*Physaloptera* spp.	1/80 (1.25) ^c^	1/100 (1) ^c^
*Cryptosporidium* spp.	11/80 (13.75) ^b^	1/100 (1) ^c^
*Strongyloides stercoralis*	-	1/100 (1) ^c^

**Table 3:** Frequencies of gastrointestinal parasites identified in wolves (W) and red foxes (RF) in the natural and anthropized areas. Significant differences in the occurrence of each recorded parasite in each area between the two wild canid species are indicated with different superscript letters.

	Natural Area		Anthropized Area	
Parasite	W (%)	RF (%)	W (%)	RF (%)
Ancylostomatidae	21/39 (53.8) ^a^	21/41 (51.2) ^a^	2/40 (5) ^b^	49/60 (81.6) ^a^
Trichuroidea	24/39 (61.5) ^a^	14/41 (34.1) ^b^	25/40 (62.5) ^a^	19/60 (31.6) ^b^
Coccidia	2/39 (5.1)	16/41 (39)	7/40 (17.5)	13/60 (21.6)
Ascarids	4/39 (10.25) ^a^	5/41 (12.2) ^a^	3/40 (7.5) ^a^	6/60 (10) ^a^
*Giardia duodenalis*	1/39 (2.56) ^b^	5/41 (12.2) ^a^	1/40 (2.5) ^a^	4/60 (6.66) ^a^
*Entamoeba* spp.	1/39 (2.56)	-	1/40 (2.5) ^a^	2/60 (3.33) ^a^
*Cryptosporidium* spp.	2/39 (5.1) ^b^	9/41 (21.9) ^a^	-	1/60 (1.66)
*Spirocerca* spp.	-	1/41 (2.43)	-	5/60 (8.33)
*Balantidium coli*	-	1/41 (2.43)	-	2/60 (3.33)
*Physaloptera* spp.	-	1/41 (2.43)	-	1/60 (1.66)
*Strongyloides stercoralis*	-	-	-	1/60 (1.66)

**Table 4:** Number of coccidian oocysts/gram of feces (OPG) and nematode eggs/gram of feces (EPG) counted in wolf and fox fecal samples collected in the anthropized (A) and natural (N) areas, respectively. Values are expressed as average ± SE (median). Significant differences are indicated with different superscript letters.

Parasite	Wolf		
	**N**	**A**	***p*** **-Value**
Ancylostomatidae	70.588 ± 43.29 ^a^	7.5 ± 3.53 ^b^	*p* < 0.0001
Trichuroidea	811.19 ± 187.194 ^a^	47.69 ± 59.77 ^b^	*p* < 0.001
Ascarids	12.12 ± 70.78 ^b^	85 ± 113.13 ^a^	*p* < 0.05
	**Red Fox**		
	**N**	**A**	***p*** **-Value**
Ancylostomatidae	148.38 ± 50.17 ^a^	139.36 ± 203.71 ^a^	*p* > 0.05
Trichuroidea	22.58 ± 218.94 ^a^	278.61 ± 703.96 ^a^	*p* > 0.05
Coccidia	403.226 ± 289.58 ^a^	153.75 ± 235.5 ^b^	*p* < 0.001
Ascarids	12.90 ± 80.58 ^b^	118 ± 114.05 ^a^	*p* < 0.0001

## Data Availability

Not applicable.
